# Long-term sero-positivity for IgG, sequelae of respiratory symptoms, and abundance of malformed sperms in a patient recovered from severe COVID-19

**DOI:** 10.1007/s10096-021-04178-6

**Published:** 2021-02-08

**Authors:** Mingchao Zhu, Diliang Chen, Ya Zhu, Xusheng Xiong, Yan Ding, Feibo Guo, Mingan Zhu, Junyang Zhou

**Affiliations:** 1 Laboratory Department, The First People’s Hospital of Tianmen City, Tianmen, 431700 Hubei China; 2 Neurology Department, The First People’s Hospital of Tianmen City, Tianmen, 431700 Hubei China; 3 Reproductive Department, The First People’s Hospital of Tianmen City, Tianmen, 431700 Hubei China; 4 Hubei Key Laboratory of Embryonic Stem Cell Research, Hubei University of Medicine, Shiyan, 442000 Hubei China; 5 Laboratory Department, Renmin Hospital, Hubei University of Medicine, Shiyan, 442000 Hubei China

**Keywords:** COVID-19, IgG, Respiratory function, Reproductive function

## Abstract

Patients with severe coronavirus disease in 2019 (COVID-19 pneumonia) may have many sequelae, which seriously affect their quality of life and work. Here, we report a case of infection in China, reviewed the course, treatment, and rehabilitation of a patient suffering from severe COVID-19 pneumonia, and collected his examination reports, including chest CT, laboratory examination results, lung function examination, sleep monitoring report, sex hormones, sperm morphology and activity. The patient’s antiviral immunoglobulin G (IgG) continued to be positive for more than 11 months, and his small airway function was abnormal, and he suffered from respiratory problems (cough, chest pain, chest tightness, and shortness of breath), unstructured sleep apnea hypopnea syndrome, and nocturnal sleep hypoxemia. His abnormal sperm rate increased obviously, and sperm activity decreased obviously. Patients with severe COVID-19 pneumonia may have respiratory sequela, the abnormal sperm rate is obviously increased, and IgG positive can last for a long time.

Since December 2019, the outbreak of pneumonia in COVID-19 caused by SARS-CoV-2 has spread all over the world. SARS-CoV-2 belongs to β-coronavirus [1], and the entry of SARS-CoV-2 into target cells may be mediated by the interaction between virus spike (s) protein and angiotensin converting enzyme 2 (ACE2) [2, 3]. Although virus transmission is mainly through respiratory droplets, SARS-CoV-2 has been isolated from blood samples and feces of patients with new crown pneumonia, which has caused problems about virus shedding in other body fluids including semen and other modes of transmission [4]. The preliminary findings of autopsy pathology reveal several conditions of SARS-CoV-2 virus particles in multiple organs, and severe patients may have many sequelae, which may seriously affect their quality of life and work [5]. Human testis is a potential target of SARS-CoV-2 infection, and the reproductive ability of pneumonia patients in COVID-19 may be significantly affected [6].

However, there are few studies on patients with severe infection, and the evidence on respiratory function and reproductive function after discharge is very limited. Here, we report a male patient with severe COVID-19 and introduce his course, treatment, and rehabilitation. Our purpose is to describe the changes of serum antibody level, respiratory function, sperm morphology, and activity of critically ill patients after discharge from hospital, so as to provide clinical evidence for further understanding the influence of COVID-19 on male respiratory function and reproductive function.

## Case presentation

The patient is a 30-year-old male. His history of exposure and exposure to COVID-19 pneumonia is unclear. Patients have no history of hypertension, diabetes or heart disease, smoking or drinking, drug allergy, respiratory diseases, and reproductive diseases. The patient was not vaccinated with SARS-CoV-2 vaccine.

**Fig. 1:**
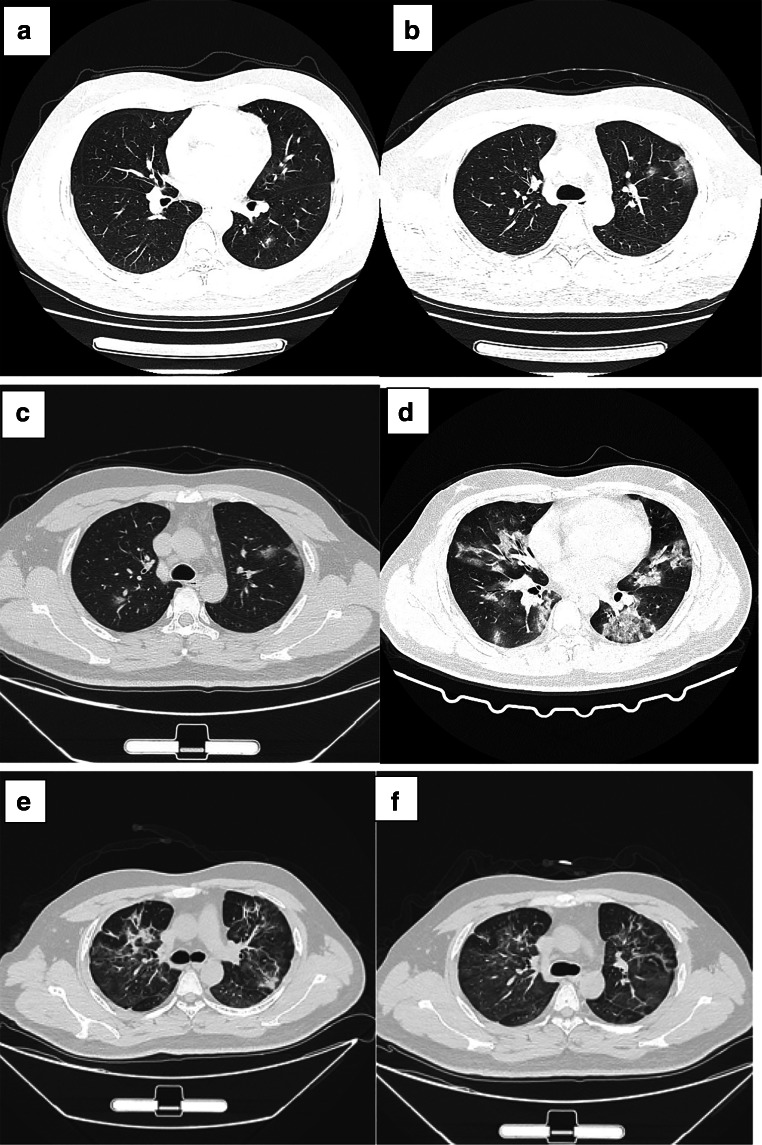

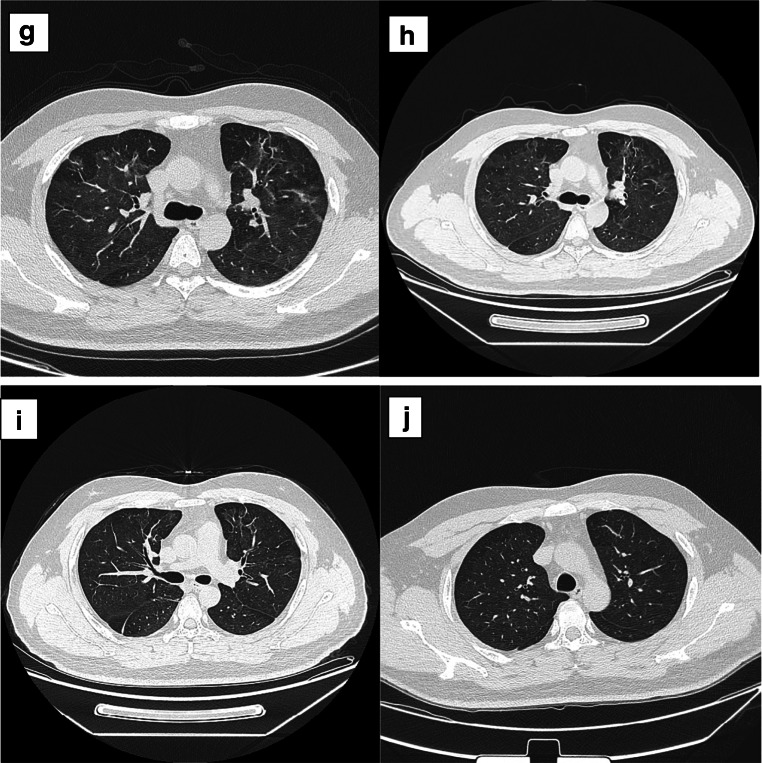
Chest CT of the patient. **a** Chest CT on January 19. **b** Chest CT on January 23. **c** Chest CT on January 25. **d** Chest CT on January 27. **e** Chest CT on February 22. **f** Chest CT on March 1. **g** Chest CT on March 9. **h** Chest CT on March 1. **i** Chest CT on April 17. **j** Chest CT on May 25

On January 19th, the patient developed fever of unknown origin with cough symptoms, mainly dry cough without chest tightness, shortness of breath, or dyspnea. His body temperature rose from 37.6 to 39.8 °C in 3 h. Chest computed tomography showed emphysema in both upper lungs, with ground glass density at the edge (Fig. 1a). The patient went home for self-isolation, but his condition did not improve, so he was transferred to the isolation area and had chest CT again on January 23th (Fig. 1b). Houkou quarantine station gave him moxifloxacin and oseltamivir for anti-infection treatment, but the effect was very little. On January 25th, he developed diarrhea 5–6 times a day, with watery stools, accompanied by fatigue, muscle pain, and anorexia. The patient was transferred to Tianmen First People’s Hospital for further treatment. On January 25th, chest CT showed infectious lesions of both lungs and localized emphysema (Fig. 1c), so he was suspected of severe viral pneumonia. On January 27th, blood test showed that C-reactive protein (CRP) was 18.16 mg/L and procalcitonin (PCT) was 0.101 ng/mL, both of which were higher than the previous tests (Table 1). On January 29th, serum immunoglobulin-G (IgG) and antiviral immunoglobulin-M (IgM) were detected by colloidal gold method (Table 2). However, the throat swab samples of SARS-CoV-2 nucleic acid on January 28th and 29th were all negative. His arterial blood oxygen saturation (SaO_2_) fluctuated around 90–94%, with symptoms of wheezing, shortness of breath, cough and sputum, and bright red blood in sputum.

**Table 1:** Laboratory test results of the infected patient

	Reference range	Jan 25	Jan 27	Jan 30	Feb 22	Mar 2	Mar 11	Jul 22
WBC (× 10^9^/L)	3.5–9.5	3.92	5.3	11.92	4.13	4.72	8.75	6.12
NEU#(× 10^9^/L)	1.8–6.3	2.23	4.0	10.99	2.02	2.05	5.65	2.93
LYM#(× 10^9^/L)	1.1–3.2	1.14	0.89	0.5	1.42	2.11	2.68	2.63
CRP (mg/L)	≤ 6	11.35	66.81	110.1	0.93	1.48	0.05	0.68
PCT (pg/mL)	≤ 0.046	0.098	0.101	0.13	< 0.020	0.028	-	-
ALT (U/L)	0–40	39.1	45.3	24.0	45.3	31.1	-	32.5
AST (U/L)	0–40	25.4	32.6	36.0	18.0	15.4	-	18.3
LDH (U/L)	120–250	242	349	245	6.2	-	-	190
HBDH (U/L)	72–182	181	284	206	480	-	-	165
CK (U/L)	50–310	158	234	71	202	-	-	189
CK-MB (U/L)	< 24	11.65	14.19	11.46	26	-	-	-
TP (g/L)	65–85	-	68.77	64.04	71.2	68.04	-	76.11
Alb (g/L)	40–55	-	43.8	38.5	28.7	41.9	-	51.8
Crea (μmol/L)	57–97	93.4	-	79.9	86.1	80.2	-	95.4
D-dimer (μg/mL)	0–0.5	-	-	Over linear	1.20	-	-	0.19
FDP (μg/mL)	0–5	-	-	2.5	3.14	-	-	0.7
FIB (g/L)	2–4	-	-	Over linear	2.93	-	-	2.43
ESR (mm/h)	0–20	-	-	68.2	-	-	-	-
SaO2 (%)	95–98	95	90	85	99	98	98	-
GM test	Negative	-	-	Positive	-	-	-	-
G test (pg/mL)	< 37.5	-	-	> 95.0	-	-	-	
MP-IgM	Negative	-	Positive	-	-	-	-	
BNP (pg/mL)	0–125	-	-	290.2	-	-	-	
Ferritin (ng/mL)	23.9–336.2	-	-	702.54	-	-	-	

*WBC* white blood cell, *NEU*# neutrophil count, *LYM#* lymphocyte count, *PLT* platelet, *CRP* C-reactive protein, *PCT* procalcitonin, *ALT* alanine aminotransferase, *AST* aspartate aminotransferase), *LDH* lactate dehydrogenase, *HBDH* hydroxybutyrate dehydrogenase, *CK* creatine kinase, *CK-MB* creatine kinase isoenzyme MB, *Crea* creatinine, *K* potassium, *Na* sodium, *Cl* chlorides, *TP* total protein, *Alb* albumin, *FDP* fibrinogen degradation products, *FIB* fibrinogen, *ESR* erythrocyte sedimentation rate, *SaO2* arterial oxygen saturation, *G test* 1,3-β-D glucan test, *GM* test galactomannan antigen detection, *MP* mycoplasma pneumoniae, *BNP* brain natriuretic petide, “-” none

**Table 2:** Detection of SARS-CoV-2 nucleic acid, IgM, and IgG antibodies

	Jan-29	Jan-30	Feb-16	Mar-20	Apr-17	May-8	May-25	Jun-19	Jul-20	Reference range(COI)	Jul-20	Aug-27	Sep-29	Oct-30	Nov-30	Dec-30
SARS CoV-2 RNA( throat swab)	-	+	-	-	-	-	-	-	-							
2019-nCoV-IgG (CGM)	+	none	none	+	+	+	+	+	+							
2019-nCoV-IgM (CGM)	+	none	none	+	-	-	-	-	-							
2019-nCoV-IgG(CLIA)										<1.0	8.17	5.84	8.30	7.87	8.12	10.37
2019-nCoV-IgM(CLIA)										<1.0	0.15	0.05	0.06	0.04	0.03	0.01

Reverse transcription-polymerase chain reaction (RT-PCR) was used for SARS-CoV-2 nucleic acid test. Colloidal gold standard method (CGM) and chemiluminescence immunoassay (CLIA) were used for 2019-nCoV-IgG and 2019-nCoV-IgG test

“-” negative, “+”positive, *COI* cut off index, reference range < 1.0 COI

As the illness worsened, the patient was immediately transferred to Wuhan Jinyintan Hospital for further treatment. On January 30th, SARS-CoV-2 nucleic acid test was positive in throat swab samples. Chest computed tomography showed ground-glass lesions in both lungs (Fig. 1d), and the blood oxygen saturation is 85–90%. In addition, routine blood analysis showed that WBC was 11.92 × 109/L, LYM was 0.5 × 10^9^/L, CRP was 110.1 mg/L, ESR was 68.2 mm/h, 1,3-β 3-β-D glucan test (G test) > 95 mg/mL, and galactomannan antigen test (GM test) was positive after a series of treatments, including extracorporeal membrane oxygenation (ECMO), anti-fungal infection (itraconazole, 0.1 g, PO, BID), anti-virus (recombinant human interferon α2b spray, 20 mL, spray, BID), anti-infection (desloratadine citrate disodium tablets, 8.880 mg, po, QD1) and glucocorticoid (methylprednisolone for injection). The throat swab test of SARS-CoV-2 nucleic acid turned negative on February 16 and 18, and then, the patient was transferred back to the First People’s Hospital of Tianmen City for follow-up treatment on February 21.

In the local hospital, chest CT showed that there were infectious lesions in both lungs on February 22th, and the lesions were partially fibrotic (Fig. 1e). Continue to give anti-virus, anti-infection and supportive treatment (the treatment scheme is the same as above), and the patient’s physical state and mental state gradually return to normal. Chest CT showed infectious lesions in both lungs. On March 1th, the lesion area was small and some lesions were fibrous (Fig. 1f). He got better and was discharged from hospital without coughing or vomiting. The patient was closely monitored at home and did not receive special treatment for 14 days. After mild exercise in isolation period, symptoms such as dizziness, chest tightness, dry cough, and shortness of breath appeared. The patient was hospitalized again on March 9th. Chest CT showed that both lungs had infectious lesions, which were smaller than the previous tests (Fig. 1g). The expert consultation of Shanxi Medical Team gave the patients TCM treatment, but the specific plan was not clear. As the above symptoms were obviously relieved, SARS-CoV-2 nucleic acid test was negative on March 9th and 11th, and he was discharged from hospital. In the next few days, he was left at home for observation and isolation. During the follow-up, he experienced chest discomfort after activities. One week later (March 20th), the patient received chest CT examination again (Fig. 1h); the results showed that the lesions were less than before. Serological tests showed that both antiviral antibodies and antiviral antibodies were positive (Table 2).

Four weeks later (April 17th), the patient’s virus-specific IgM became negative, but IgG was still positive (Table 2). On April 17th, chest CT was reexamined, showing that the area of lung lesions decreased and the density increased compared with before (Fig. 1i). The serological test results of IgG and IgM in patients on May 8 and May 25 were the same as last time (Table 2). On May 25th, chest CT examination showed that there were many strip-shaped and sheet-shaped high-density lesions in both lungs, accompanied by pleural adhesion; the lesion scope was smaller than before, and the partial density decreased (Fig. 1j). On July 20th, patients’ IgG was still positive (Table 2), and the cut-off index (COI) value of IgG detected by chemiluminescence immunoassay (CLIA) was 8.17 (reference range < 1.0 COI). During the follow-up, the antibody was continuously positive (Table 2).

Because of chest tightness and chest pain after discharge, and respiratory disturbance after exercise, we performed pulmonary function examination and sleep monitoring. According to the sleep monitoring report on May 31th, the patient’s apnea hypopnea index (AHI) was 8.53 (normal < 5.0), and his blood oxygen saturation during sleep was 92% (reference range: 94–98%). He was diagnosed as obstructive sleep apnea hypopnea syndrome and nocturnal sleep hypoxemia (Table 3), which did not exist before COVID-19 pneumonia infection. Lung function test showed that forced expiratory flow 75 (FEF75), maximum expiratory flow (MMEF), and mean effective pressure (MEP) of the patient decreased sharply, and his forced vital capacity (FVC), forced expiratory volume for 1 s (FEV1), and maximum expiratory flow (PEF) also decreased. The pulmonary ventilation function was normal, but the small airway function was abnormal (Table 3). In daily life, the patient has cough, chest pain, chest tightness, and other symptoms from time to time, and these symptoms did not disappear until November.

**Table 3:** The test results of pulmonary function test, sleep monitoring report

	Reference range	Mar 28
FEV1 (L)	3.245	2.656
VCmax (L)	3.907	3.494
FVC (L)	3.907	3.399
PEF (L/s)	9.41	7.84
IC (L)	2.923	2.554
FEF25 (L/s)	7.5	7.86
FEF50 (L/s)	4.37	3.55
FEF75 (L/s)	1.82	0.67
MMEF	4.07	2.31
MEP (cmH_2_O)	237	156
MIP (cmH_2_O)	126	80
MV (L)	10.714	8.161
ERV (L)	1.408	0.793
Apnea hypopnea index	< 5	8.53
Oxyhemoglobin saturation(%)	95–98	92

*FEF* forced expiratory flow, *MMEF* maximum mid-expiratory flow, *MEP* mean effective pressure, *MIP* maximum inspiratory pressure, *MV* minute ventilation, *ERV* expiratory reserve volume, *FEV1*forced expiratory volume in one second, *VC* vital capacity, *FVC* forced vital capacity, *PEF* peak expiratory flow, *IC* inspiratory capacity

**Fig. 2:**
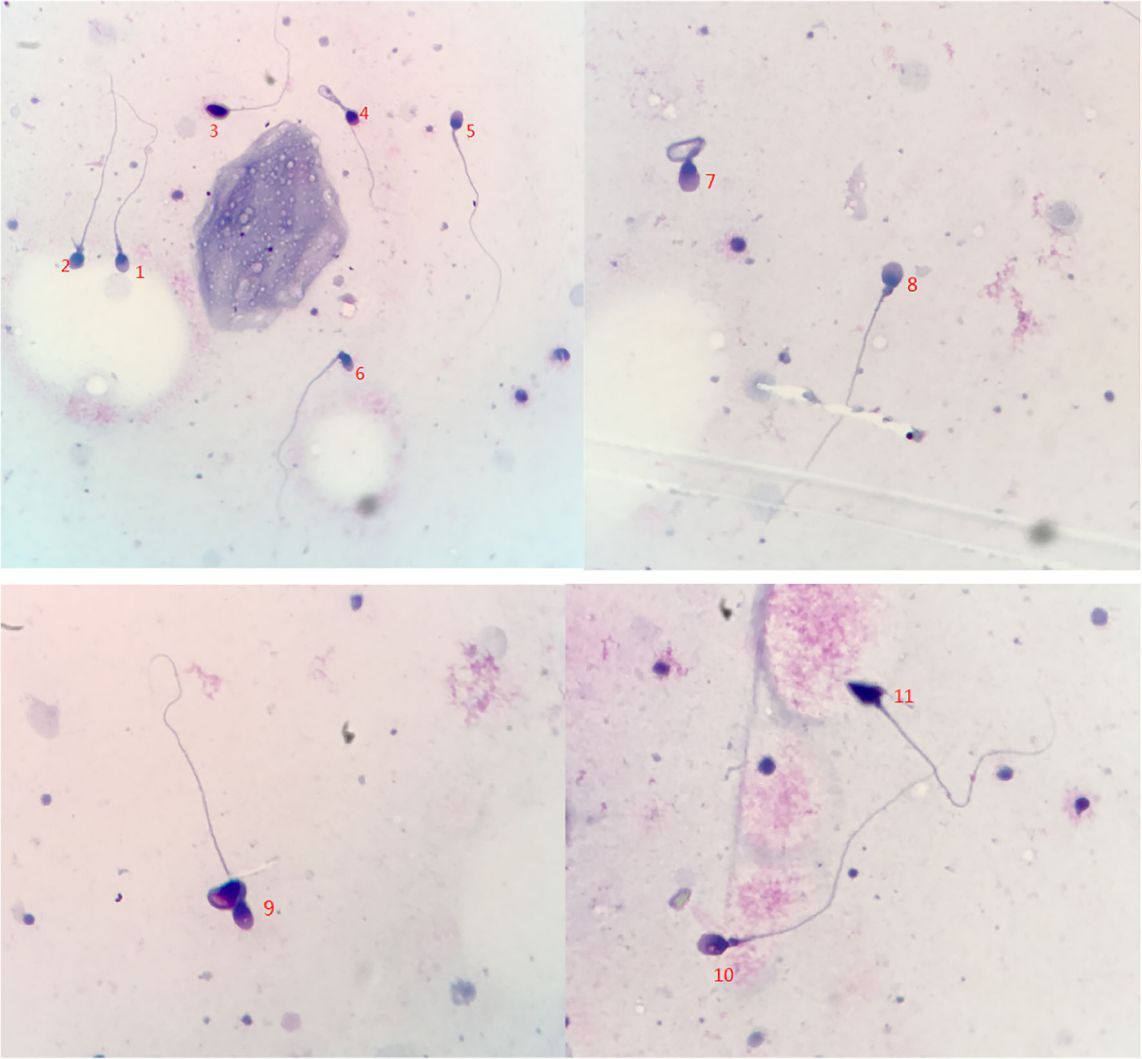
Sperm appeared various deformities. Sperm morphology detection conducted by Diff Quick staining. (1) Normal, (2) round, (3) thick, (4) Coiled, (5) normal, (6) bent neck, (7) coiled, (8) cytoplasm, (9) double, (10) PA vac, (11) tapered

On July 20th, SARS-CoV-2 nucleic acid was not detected by reverse transcription polymerase chain reaction (RT-PCR) in patients’ sperm. In order to study the influence of COVID-19 pneumonia on the fertility of the patient, we collected his semen and examined the morphology and activity of sperm. To our surprise, out of 227 sperm, 219 sperm were deformed (96.48%), and the head deformity, middle deformity, and tail deformity were 96.47%, 35.68%, and 9.69%, respectively (Table 4). The morphology of abnormal sperm is shown in Fig. 2. Sperm motility test showed that non-progressive (NP) sperm accounted for 18.5%, progressive (PR) sperm accounted for 25.0%, immobile (IM) sperm accounted for 56.5%, sperm concentration was 7.9 × 10^6^/mL (normal > 15 × 10^6^/mL), and only 11 sperm could move in a straight line (observed sperm). Curve velocity (VCL), straight line velocity (VSL), average path velocity (VAP), and linearity (forest) have obvious downward trend (Table 4). Sex hormone test showed that estradiol was slightly higher (17.530 ng/mL, reference range: 4.04–15.2 ng/mL), while luteinizing hormone (LH), follicle stimulating hormone (FSH), estradiol, and testosterone were normal (Table 4).

**Table 4:** The test results of sex hormone, the morphology, and activity of sperm

	Reference range	Results
Sperm morphology report		
Jun 22		
Total sperm number analyzed		227
Normal sperm (%)	4	3.52
Malformed sperm rate(%)		96.48
Total number of abnormal		322
Head malformation (%)		96.47
Neck and midpiece malformation (%)		35.68
Tail malformation (%)		9.69
Sex hormone test		
LH (mIU/mL)	1.7–8.6	6.23
FSH (mIU/mL)	1.5–12.4	4.95
Estradiol (pg/mL)	11.3–43.2	24.21
Prolactin (ng/mL)	4.05–15.2	17.53
Testosterone (ng/mL)	2.49–8.36	3.26
Sperm activity		
Jul 26		
pH	≥ 7.2	7.8
Semen volume (mL)	≥ 1.5	3.5
Liquefaction time (min)	≤ 60	15
Sperm concentration (× 106/mL)	≥ 15	7.9
PR+NP (%)	≥ 40	43.5
PR%	≥ 32.0	25.0
NP%	1	18.5
IM%	22	56.5
VAP (mm/s)		26.6
VCL (mm/s)		41.8
VSL (mm/s)		22.7
LIN (%)		42.6
Survival rate (%) (eosin-aniline black stain)	≥ 58	80

*NP* non-progressive, *PR* progressive, *IM* immotility, *VCL* curvilinear velocity, *VSL* straight-line velocity, *VAP* average path velocity, *LIN* linearity

## Discussion

Patients with SARS-CoV-2 infection may have different symptoms [7, 8]. However, we know little about the clinical features and rehabilitation of COVID-19 critically ill patients. Here, we describe a patient infected with SARS-CoV-2, who rapidly developed into severe COVID-19. We reviewed his course of disease, treatment, and recovery and found that the sustained positive IgG lasted for an astonishing 11 months, which should be the longest duration reported so far. After he was discharged from hospital, his life and work were still affected by persistent respiratory problems. His abnormal sperm rate increased significantly and sperm activity decreased significantly, but we did not detect viral nucleic acid in his semen.

On January 19th, the patient developed fever and dry cough, and chest CT showed frosted glass opacity (Fig. 1a), followed by diarrhea. After admission, the illness worsened, blood oxygen saturation decreased (90–94%), and acute respiratory distress syndrome (ARDS) occurred. He was transferred to Wuhan Jinyintan Hospital on January 29th. Subsequently, he was diagnosed as COVID-19 and received ECMO treatment, and the symptoms of hypoxia quickly eased. G test and GM test of patients were positive (Table 1), suggesting fungal infection accompanied by the increase of inflammatory markers such as CRP and PCT (Table 1). The patient developed combined immunodeficiency, which may be caused by cytokine storm. Cytokine storm is an excessive immune response to external stimuli [9]. Cytokine storm is considered as one of the main causes of acute respiratory distress syndrome and multiple organ failure in COVID-19 patients [9, 10]. Therefore, effective suppression of cytokine storm is the key to prevent COVID-19 infection from worsening. Previous studies suggested screening patients with severe COVID-19 for high inflammation caused by virus and suggested that immunomodulation may reduce the high mortality rate in this group [11, 12]. After antiviral, antifungal, and glucocorticoid treatment, his condition has improved obviously, which may be related to the effective suppression of cytokine storm. SARS-CoV-2 nucleic acid was negative after reexamination, and the patient was discharged on March 7th.

Some studies have shown that COVID-19 pneumonia virus-specific antibody detection can be used as an important supplement to nucleic acid detection, to diagnose suspected cases with negative RT-PCR results, and to investigate asymptomatic infections of close contacts [13]. IgM and IgG antibodies may be positive as early as the 4th day after onset [14]. Long et al. reported that 100% patients detected antibody positive within 19 days after symptoms appeared [15]. The research in Iceland also showed that the anti-virus antibody against SARS-CoV-2 did not decrease within 5 months after diagnosis [15]. In this case, we tested IgG and IgM 10 days after fever, and both were positive. On April 17th, his virus-specific IgM was negative, but IgG was still positive. In our recent serological test (December 30th), the antibody was still positive and the level remained stable (Table 3), so we speculated that the duration of antibody positive was more than 11 months, which will be the longest duration of antibody so far. It is not clear whether human infection with SARS-CoV-2 can prevent re-infection and how long it will last [16], but many studies in vivo and in vitro show that even if an individual is re-infected, antibodies can prevent re-infection or alleviate the disease [17–21]. Although our case could not provide conclusive evidence that these antibody reactions can prevent reinfection, it will provide a better understanding of SARS-CoV-2 antibody and may be beneficial to vaccine development.

It is generally believed that the severity of the disease is an independent predictor of poor prognosis of pneumonia in COVID-19 [22, 23]. Studies have shown that SARS-COV-2 infection can lead to multiple organ and tissue injuries, accompanied by significant and extensive lung diseases [24–26]. Patients who have been admitted to intensive care unit (ICU) will have more serious complications, especially those who have been treated by ventilator, and their lungs, brain, heart, muscles, and nervous system will have sequelae [5, 27–31]. In this case, the patient has some respiratory problems after discharge, such as cough, fatigue, and chest pain after exercise, and chest tightness and shortness of breath may occur when walking fast. He returned to work on May 26th, but he can only take on some simple and easy work. According to the sleep monitoring report, the patient suffered from obstructive sleep apnea hypopnea syndrome and nocturnal sleep hypoxemia (Table 3). His small airway function was abnormal, but many respiratory function indexes decreased (Table 3). He did not have these problems before being attacked by COVID-19 pneumonia. These respiratory problems obviously affected the normal life of patients and disappeared slowly after 11 months.

ACE2 is the main receptor of SARS-CoV-2, which is highly expressed in testis [24]. In Fu's research, SARS-CoV-2 was not detected in the semen of recovered patients 1 month after diagnosis [32]. On the contrary, a study by the General Hospital of Chinese People’s Liberation Army found that SARS-CoV-2 was detected in semen of both acute and convalescent patients [33], but this study did not show how much viral load there was in semen, whether it was a residual fragment or a complete virus particle and could not confirm whether it was contagious. In this study, the patient gave birth to a healthy girl a year ago, who had no history of drug allergy, reproductive system diseases, and had not been vaccinated with SARS-CoV-2 vaccine. No viral nucleic acid was detected in his semen. The decrease of sperm survival rate and sperm motility is an important reason for male infertility, and sperm deformity will also affect male reproductive function. Six months after SARS-CoV-2 infection, we tested the sperm of this patient, the results showed that the abnormal sperm rate was as high as 96.48%, the sperm activity decreased sharply (Table 4), and most of the sperm were deformed in the head, body, and tail (Fig. 2). He was diagnosed with teratospermia. A single-center-based study compared the sex-related hormones between 81 reproductive-age men infected with SARS-CoV-2 and 100 age-matched healthy men; it was found that the serum LH of COVID-19 pneumonia men increased significantly, but the ratio of testosterone to LH and follicle stimulating hormone to luteinizing hormone decreased significantly. Therefore, the infection of COVID-19 pneumonia might seriously damage the male sexual development of young men and lead to infertility of adult men [34]. In this case, the patient’s estradiol is slightly higher, but his luteinizing hormone, follicle stimulating hormone, estradiol, and testosterone are normal (Table 4), which was not completely consistent with previous studies.

In conclusion, our findings show that in severe patients, IgG can remain positive for a long time, the recovery period of respiratory function may be longer, and a large number of abnormal sperm may appear in patients who have recovered from severe pneumonia, while the sperm of recovered patients may not be contagious. Of course, it should not underestimate the possible side effects of antiviral drugs, glucocorticoids, or traditional Chinese medicine in the treatment of COVID-19 pneumonia, which may have systemic effects on some patients and lead to infertility. Because the number of cases is insufficient, our research does not provide conclusive evidence, nor can we infer all cases. Therefore, it is imperative to quickly carry out research and investigation and carry out long-term monitoring and follow-up for the critically ill patients after rehabilitation. The struggle between mankind and plague will not end.

## Data Availability

No additional data are available.
